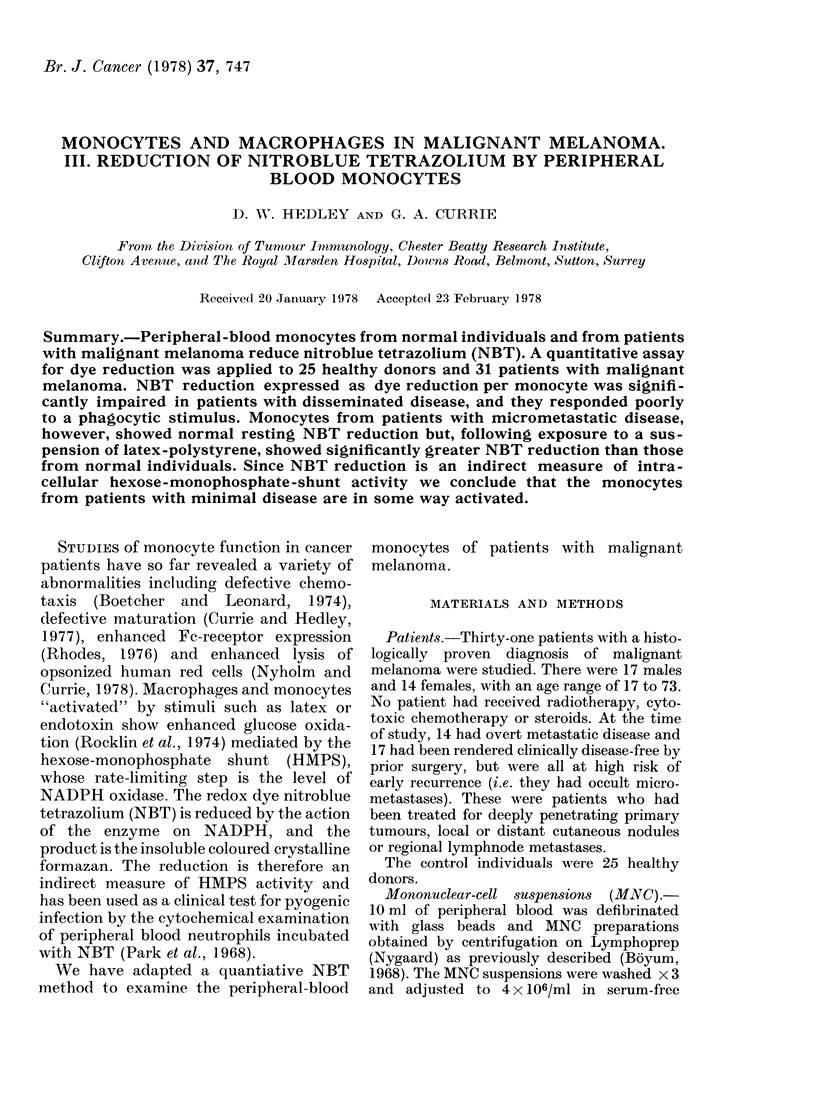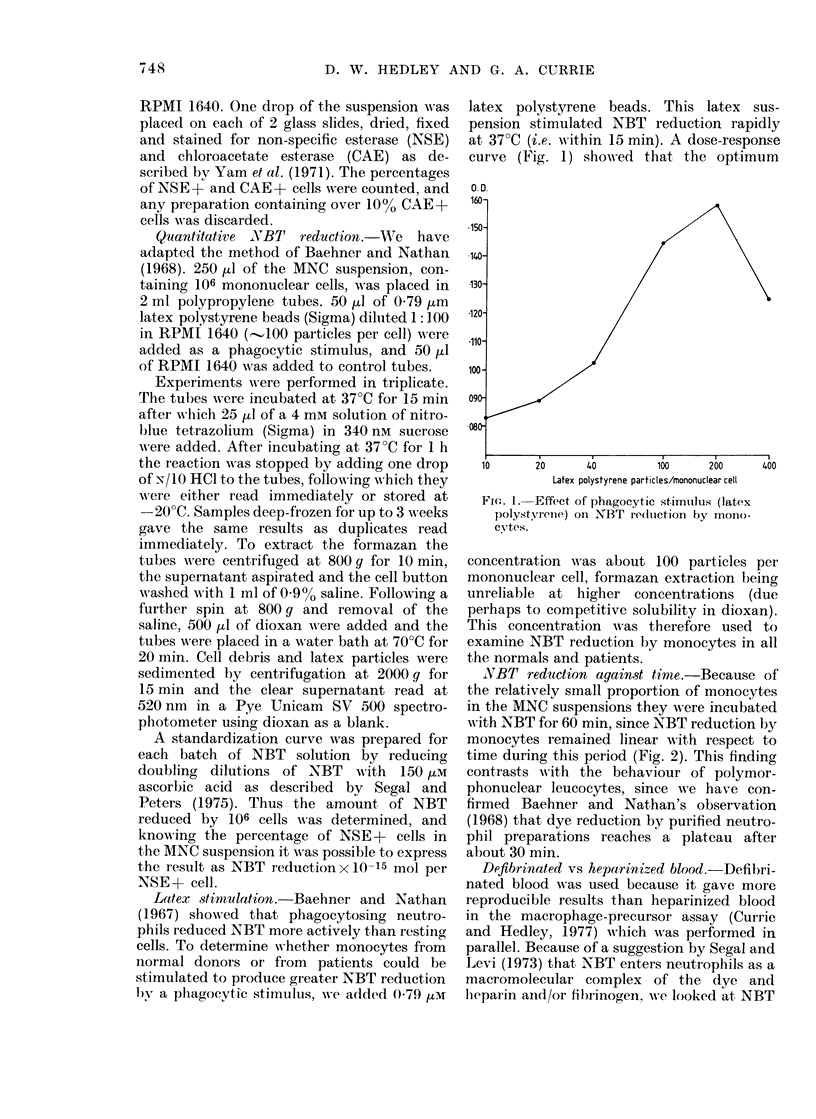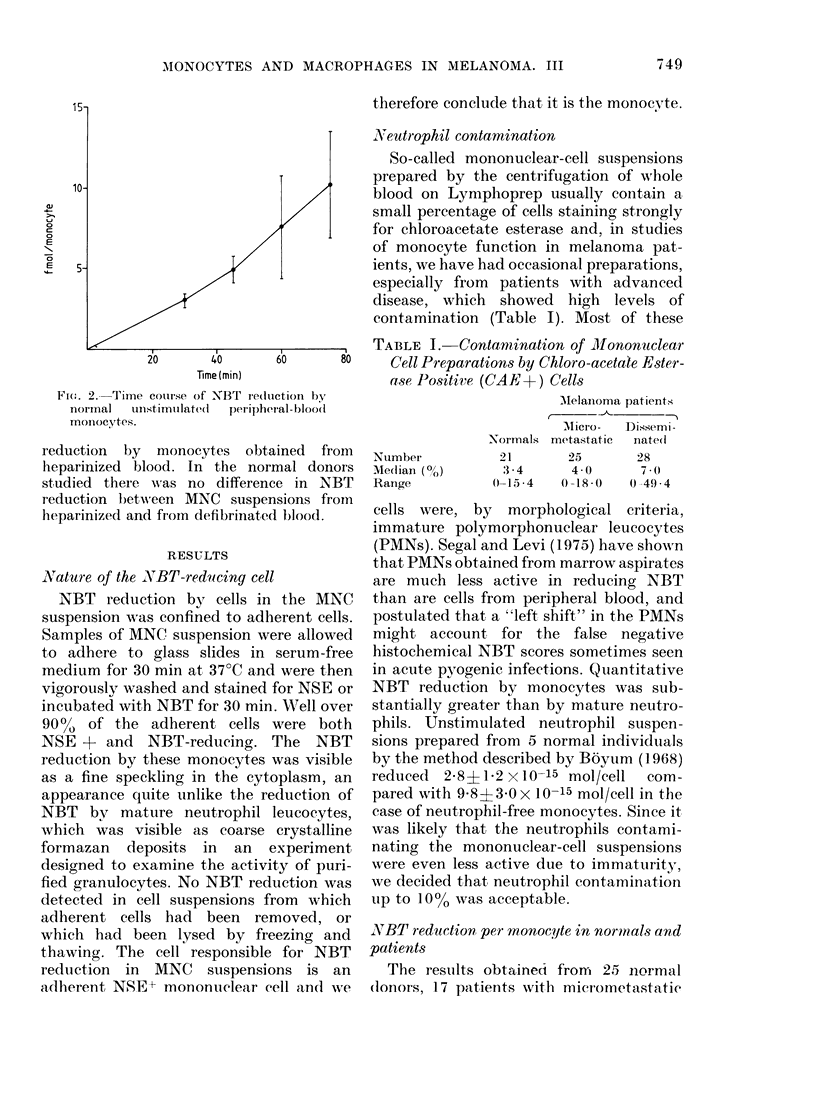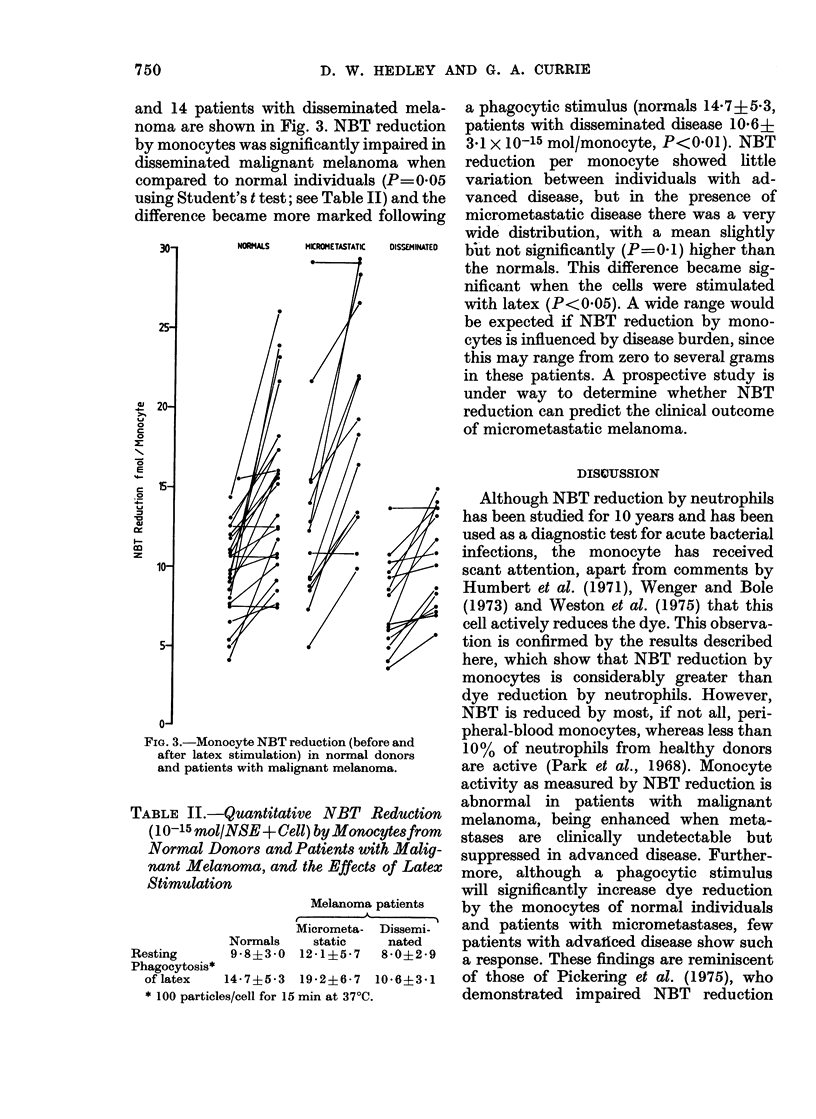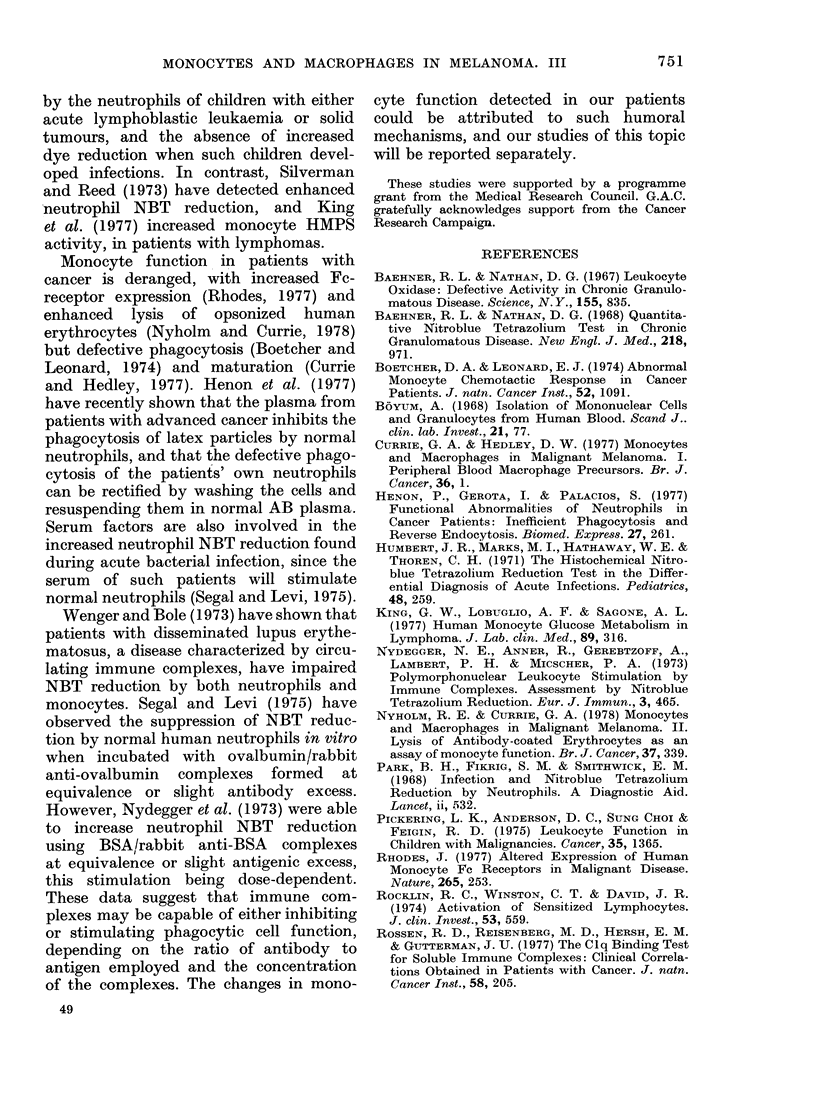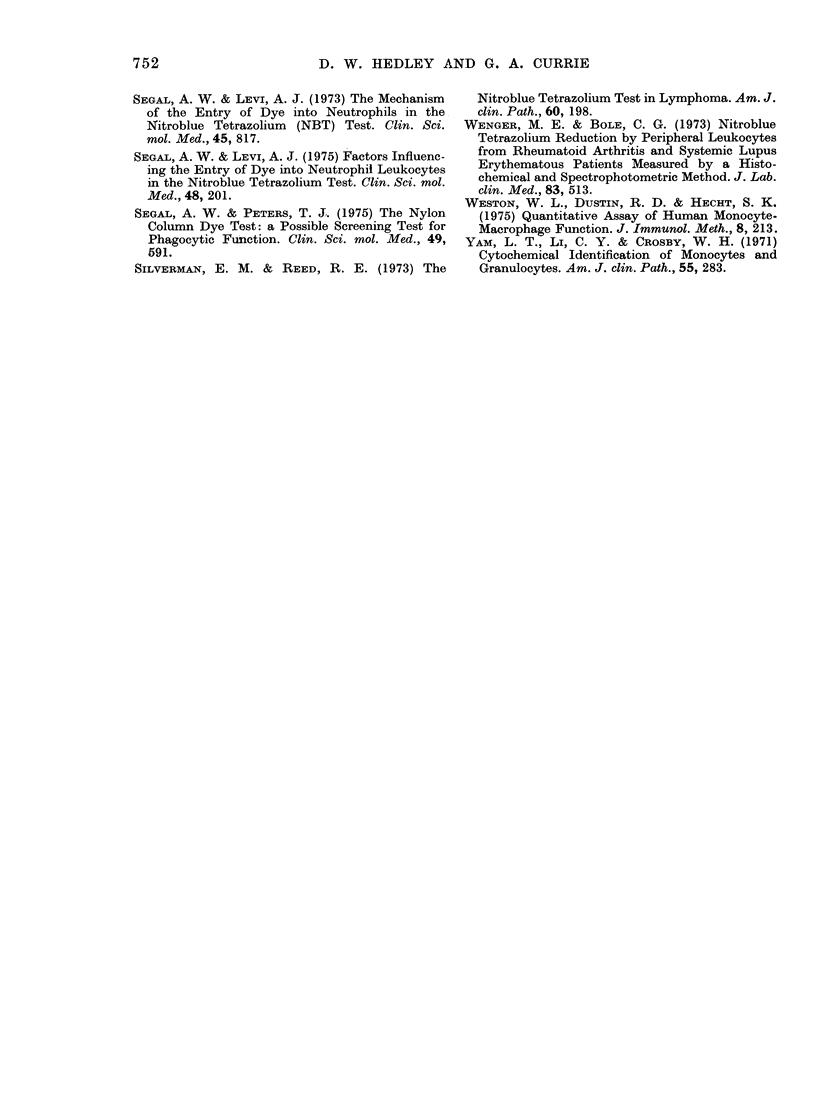# Monocytes and macrophages in malignant melanoma. III. Reduction of nitroblue tetrazolium by peripheral blood monocytes.

**DOI:** 10.1038/bjc.1978.113

**Published:** 1978-05

**Authors:** D. W. Hedley, G. A. Currie

## Abstract

Peripheral-blood monocytes from normal individuals and from patients with malignant melanoma reduce nitroblue tetrazolium (NBT). A quantitative assay for dye reduction was applied to 25 healthy donors and 31 patients with malignant melanoma. NBT reduction expressed as dye reduction per monocyte was significantly impaired in patients with disseminated disease, and they responded poorly to a phagocytic stimulus. Monocytes from patients with micrometastatic disease, however, showed normal resting NBT reduction but, following exposure to a suspension of latex-polystyrene, showed significantly greater NBT reduction than those from normal individuals. Since NBT reduction is an indirect measure of intracellular hexose-monophosphate-shunt activity we conclude that the monocytes from patients with minimal disease are in some way activated.


*Br. J. Cancer* (1978) **37**, 747

### MONOCYTES AND MACROPHAGES IN MALIGNANT MELANOMA. III. REDUCTION OF NITROBLUE TETRAZOLIUM BY PERIPHERAL BLOOD MONOCYTES

D. W. HEDLEY AND G. A. CURRIE

*From the Division of Tumour Immunology, Chester Beatty Research Institute, Clifton Avenue, and The Royal Marsden Hospital, Downs Road, Belmont, Sutton, Surrey*

Received 20 January 1978 Accepted 23 February 1978

**Summary.—Peripheral-blood monocytes from normal individuals and from patients with malignant melanoma reduce nitroblue tetrazolium (NBT). A quantitative assay for dye reduction was applied to 25 healthy donors and 31 patients with malignant melanoma. NBT reduction expressed as dye reduction per monocyte was significantly impaired in patients with disseminated disease, and they responded poorly to a phagocytic stimulus. Monocytes from patients with micrometastatic disease, however, showed normal resting NBT reduction but, following exposure to a suspension of latex-polystyrene, showed significantly greater NBT reduction than those from normal individuals. Since NBT reduction is an indirect measure of intracellular hexose-monophosphate-shunt activity we conclude that the monocytes from patients with minimal disease are in some way activated.**

STUDIES of monocyte function in cancer patients have so far revealed a variety of abnormalities including defective chemotaxis (Boetcher and Leonard, 1974), defective maturation (Currie and Hedley, 1977), enhanced Fc-receptor expression (Rhodes, 1976) and enhanced lysis of opsonized human red cells (Nyholm and Currie, 1978). Macrophages and monocytes "activated" by stimuli such as latex or endotoxin show enhanced glucose oxidation (Rocklin *et al.*, 1974) mediated by the hexose-monophosphate shunt (HMPS), whose rate-limiting step is the level of NADPH oxidase. The redox dye nitroblue tetrazolium (NBT) is reduced by the action of the enzyme on NADPH, and the product is the insoluble coloured crystalline formazan. The reduction is therefore an indirect measure of HMPS activity and has been used as a clinical test for pyogenic infection by the cytochemical examination of peripheral blood neutrophils incubated with NBT (Park *et al.*, 1968).

We have adapted a quantiative NBT method to examine the peripheral-blood monocytes of patients with malignant melanoma.

### MATERIALS AND METHODS

*Patients.*—Thirty-one patients with a histologically proven diagnosis of malignant melanoma were studied. There were 17 males and 14 females, with an age range of 17 to 73. No patient had received radiotherapy, cytotoxic chemotherapy or steroids. At the time of study, 14 had overt metastatic disease and 17 had been rendered clinically disease-free by prior surgery, but were all at high risk of early recurrence (*i.e.* they had occult micrometastases). These were patients who had been treated for deeply penetrating primary tumours, local or distant cutaneous nodules or regional lymphnode metastases.

The control individuals were 25 healthy donors.

*Mononuclear-cell suspensions (MNC).*—10 ml of peripheral blood was defibrinated with glass beads and MNC preparations obtained by centrifugation on Lymphoprep (Nygaard) as previously described (Böyum, 1968). The MNC suspensions were washed $\times 3$ and adjusted to $4 \times 10^6$/ml in serum-free

RPMI 1640. One drop of the suspension was placed on each of 2 glass slides, dried, fixed and stained for non-specific esterase (NSE) and chloroacetate esterase (CAE) as described by Yam *et al.* (1971). The percentages of NSE+ and CAE+ cells were counted, and any preparation containing over 10% CAE+ cells was discarded.

*Quantitative NBT reduction.*—We have adapted the method of Baehner and Nathan (1968). 250 μl of the MNC suspension, containing $10^6$ mononuclear cells, was placed in 2 ml polypropylene tubes. 50 μl of 0·79 μm latex polystyrene beads (Sigma) diluted 1:100 in RPMI 1640 (~100 particles per cell) were added as a phagocytic stimulus, and 50 μl of RPMI 1640 was added to control tubes.

Experiments were performed in triplicate. The tubes were incubated at 37°C for 15 min after which 25 μl of a 4 mM solution of nitroblue tetrazolium (Sigma) in 340 nM sucrose were added. After incubating at 37°C for 1 h the reaction was stopped by adding one drop of N/10 HCl to the tubes, following which they were either read immediately or stored at −20°C. Samples deep-frozen for up to 3 weeks gave the same results as duplicates read immediately. To extract the formazan the tubes were centrifuged at 800 *g* for 10 min, the supernatant aspirated and the cell button washed with 1 ml of 0·9% saline. Following a further spin at 800 *g* and removal of the saline, 500 μl of dioxan were added and the tubes were placed in a water bath at 70°C for 20 min. Cell debris and latex particles were sedimented by centrifugation at 2000 *g* for 15 min and the clear supernatant read at 520 nm in a Pye Unicam SV 500 spectrophotometer using dioxan as a blank.

A standardization curve was prepared for each batch of NBT solution by reducing doubling dilutions of NBT with 150 μM ascorbic acid as described by Segal and Peters (1975). Thus the amount of NBT reduced by $10^6$ cells was determined, and knowing the percentage of NSE+ cells in the MNC suspension it was possible to express the result as NBT reduction $\times 10^{-15}$ mol per NSE+ cell.

*Latex stimulation.*—Baehner and Nathan (1967) showed that phagocytosing neutrophils reduced NBT more actively than resting cells. To determine whether monocytes from normal donors or from patients could be stimulated to produce greater NBT reduction by a phagocytic stimulus, we added 0·79 μM latex polystyrene beads. This latex suspension stimulated NBT reduction rapidly at 37°C (*i.e.* within 15 min). A dose-response curve (Fig. 1) showed that the optimum

FIG. 1.—Effect of phagocytic stimulus (latex polystyrene) on NBT reduction by monocytes.

concentration was about 100 particles per mononuclear cell, formazan extraction being unreliable at higher concentrations (due perhaps to competitive solubility in dioxan). This concentration was therefore used to examine NBT reduction by monocytes in all the normals and patients.

*NBT reduction against time.*—Because of the relatively small proportion of monocytes in the MNC suspensions they were incubated with NBT for 60 min, since NBT reduction by monocytes remained linear with respect to time during this period (Fig. 2). This finding contrasts with the behaviour of polymorphonuclear leucocytes, since we have confirmed Baehner and Nathan's observation (1968) that dye reduction by purified neutrophil preparations reaches a plateau after about 30 min.

*Defibrinated* vs *heparinized blood.*—Defibrinated blood was used because it gave more reproducible results than heparinized blood in the macrophage-precursor assay (Currie and Hedley, 1977) which was performed in parallel. Because of a suggestion by Segal and Levi (1973) that NBT enters neutrophils as a macromolecular complex of the dye and heparin and/or fibrinogen, we looked at NBT

FIG. 2.—Time course of NBT reduction by normal unstimulated peripheral-blood monocytes.

reduction by monocytes obtained from heparinized blood. In the normal donors studied there was no difference in NBT reduction between MNC suspensions from heparinized and from defibrinated blood.

### RESULTS

*Nature of the NBT-reducing cell*

NBT reduction by cells in the MNC suspension was confined to adherent cells. Samples of MNC suspension were allowed to adhere to glass slides in serum-free medium for 30 min at 37°C and were then vigorously washed and stained for NSE or incubated with NBT for 30 min. Well over 90% of the adherent cells were both NSE + and NBT-reducing. The NBT reduction by these monocytes was visible as a fine speckling in the cytoplasm, an appearance quite unlike the reduction of NBT by mature neutrophil leucocytes, which was visible as coarse crystalline formazan deposits in an experiment designed to examine the activity of purified granulocytes. No NBT reduction was detected in cell suspensions from which adherent cells had been removed, or which had been lysed by freezing and thawing. The cell responsible for NBT reduction in MNC suspensions is an adherent $NSE^+$ mononuclear cell and we therefore conclude that it is the monocyte.

*Neutrophil contamination*

So-called mononuclear-cell suspensions prepared by the centrifugation of whole blood on Lymphoprep usually contain a small percentage of cells staining strongly for chloroacetate esterase and, in studies of monocyte function in melanoma patients, we have had occasional preparations, especially from patients with advanced disease, which showed high levels of contamination (Table I). Most of these cells were, by morphological criteria, immature polymorphonuclear leucocytes (PMNs). Segal and Levi (1975) have shown that PMNs obtained from marrow aspirates are much less active in reducing NBT than are cells from peripheral blood, and postulated that a "left shift" in the PMNs might account for the false negative histochemical NBT scores sometimes seen in acute pyogenic infections. Quantitative NBT reduction by monocytes was substantially greater than by mature neutrophils. Unstimulated neutrophil suspensions prepared from 5 normal individuals by the method described by Böyum (1968) reduced $2{\cdot}8 \pm 1{\cdot}2 \times 10^{-15}$ mol/cell compared with $9{\cdot}8 \pm 3{\cdot}0 \times 10^{-15}$ mol/cell in the case of neutrophil-free monocytes. Since it was likely that the neutrophils contaminating the mononuclear-cell suspensions were even less active due to immaturity, we decided that neutrophil contamination up to 10% was acceptable.

TABLE I.—*Contamination of Mononuclear Cell Preparations by Chloro-acetate Esterase Positive (CAE+) Cells*

| | | Melanoma patients | |
|---|---|---|---|
| | Normals | Micro-metastatic | Disseminated |
| Number | 21 | 25 | 28 |
| Median (%) | 3·4 | 4·0 | 7·0 |
| Range | 0–15·4 | 0–18·0 | 0–49·4 |

*NBT reduction per monocyte in normals and patients*

The results obtained from 25 normal donors, 17 patients with micrometastatic and 14 patients with disseminated melanoma are shown in Fig. 3. NBT reduction by monocytes was significantly impaired in disseminated malignant melanoma when compared to normal individuals ($P = 0{\cdot}05$ using Student's $t$ test; see Table II) and the difference became more marked following a phagocytic stimulus (normals $14{\cdot}7 \pm 5{\cdot}3$, patients with disseminated disease $10{\cdot}6 \pm 3{\cdot}1 \times 10^{-15}$ mol/monocyte, $P < 0{\cdot}01$). NBT reduction per monocyte showed little variation between individuals with advanced disease, but in the presence of micrometastatic disease there was a very wide distribution, with a mean slightly but not significantly ($P = 0{\cdot}1$) higher than the normals. This difference became significant when the cells were stimulated with latex ($P < 0{\cdot}05$). A wide range would be expected if NBT reduction by monocytes is influenced by disease burden, since this may range from zero to several grams in these patients. A prospective study is under way to determine whether NBT reduction can predict the clinical outcome of micrometastatic melanoma.

FIG. 3.—Monocyte NBT reduction (before and after latex stimulation) in normal donors and patients with malignant melanoma.

TABLE II.—*Quantitative NBT Reduction ($10^{-15}$ mol/NSE + Cell) by Monocytes from Normal Donors and Patients with Malignant Melanoma, and the Effects of Latex Stimulation*

| | | Melanoma patients | |
|---|---|---|---|
| | Normals | Micrometastatic | Disseminated |
| Resting | 9·8±3·0 | 12·1±5·7 | 8·0±2·9 |
| Phagocytosis* of latex | 14·7±5·3 | 19·2±6·7 | 10·6±3·1 |

* 100 particles/cell for 15 min at 37°C.

### DISCUSSION

Although NBT reduction by neutrophils has been studied for 10 years and has been used as a diagnostic test for acute bacterial infections, the monocyte has received scant attention, apart from comments by Humbert *et al.* (1971), Wenger and Bole (1973) and Weston *et al.* (1975) that this cell actively reduces the dye. This observation is confirmed by the results described here, which show that NBT reduction by monocytes is considerably greater than dye reduction by neutrophils. However, NBT is reduced by most, if not all, peripheral-blood monocytes, whereas less than 10% of neutrophils from healthy donors are active (Park *et al.*, 1968). Monocyte activity as measured by NBT reduction is abnormal in patients with malignant melanoma, being enhanced when metastases are clinically undetectable but suppressed in advanced disease. Furthermore, although a phagocytic stimulus will significantly increase dye reduction by the monocytes of normal individuals and patients with micrometastases, few patients with advanced disease show such a response. These findings are reminiscent of those of Pickering *et al.* (1975), who demonstrated impaired NBT reduction by the neutrophils of children with either acute lymphoblastic leukaemia or solid tumours, and the absence of increased dye reduction when such children developed infections. In contrast, Silverman and Reed (1973) have detected enhanced neutrophil NBT reduction, and King *et al.* (1977) increased monocyte HMPS activity, in patients with lymphomas.

Monocyte function in patients with cancer is deranged, with increased Fc-receptor expression (Rhodes, 1977) and enhanced lysis of opsonized human erythrocytes (Nyholm and Currie, 1978) but defective phagocytosis (Boetcher and Leonard, 1974) and maturation (Currie and Hedley, 1977). Henon *et al.* (1977) have recently shown that the plasma from patients with advanced cancer inhibits the phagocytosis of latex particles by normal neutrophils, and that the defective phagocytosis of the patients' own neutrophils can be rectified by washing the cells and resuspending them in normal AB plasma. Serum factors are also involved in the increased neutrophil NBT reduction found during acute bacterial infection, since the serum of such patients will stimulate normal neutrophils (Segal and Levi, 1975).

Wenger and Bole (1973) have shown that patients with disseminated lupus erythematosus, a disease characterized by circulating immune complexes, have impaired NBT reduction by both neutrophils and monocytes. Segal and Levi (1975) have observed the suppression of NBT reduction by normal human neutrophils *in vitro* when incubated with ovalbumin/rabbit anti-ovalbumin complexes formed at equivalence or slight antibody excess. However, Nydegger *et al.* (1973) were able to increase neutrophil NBT reduction using BSA/rabbit anti-BSA complexes at equivalence or slight antigenic excess, this stimulation being dose-dependent. These data suggest that immune complexes may be capable of either inhibiting or stimulating phagocytic cell function, depending on the ratio of antibody to antigen employed and the concentration of the complexes. The changes in monocyte function detected in our patients could be attributed to such humoral mechanisms, and our studies of this topic will be reported separately.

These studies were supported by a programme grant from the Medical Research Council. G.A.C. gratefully acknowledges support from the Cancer Research Campaign.

### REFERENCES

BAEHNER, R. L. & NATHAN, D. G. (1967) Leukocyte Oxidase: Defective Activity in Chronic Granulomatous Disease. *Science, N.Y.*, **155**, 835.

BAEHNER, R. L. & NATHAN, D. G. (1968) Quantitative Nitroblue Tetrazolium Test in Chronic Granulomatous Disease. *New Engl. J. Med.*, **218**, 971.

BOETCHER, D. A. & LEONARD, E. J. (1974) Abnormal Monocyte Chemotactic Response in Cancer Patients. *J. natn. Cancer Inst.*, **52**, 1091.

BÖYUM, A. (1968) Isolation of Mononuclear Cells and Granulocytes from Human Blood. *Scand J.. clin. lab. Invest.*, **21**, 77.

CURRIE, G. A. & HEDLEY, D. W. (1977) Monocytes and Macrophages in Malignant Melanoma. I. Peripheral Blood Macrophage Precursors. *Br. J. Cancer*, **36**, 1.

HENON, P., GEROTA, I. & PALACIOS, S. (1977) Functional Abnormalities of Neutrophils in Cancer Patients: Inefficient Phagocytosis and Reverse Endocytosis. *Biomed. Express.* **27**, 261.

HUMBERT, J. R., MARKS, M. I., HATHAWAY, W. E. & THOREN, C. H. (1971) The Histochemical Nitroblue Tetrazolium Reduction Test in the Differential Diagnosis of Acute Infections. *Pediatrics*, **48**, 259.

KING, G. W., LOBUGLIO, A. F. & SAGONE, A. L. (1977) Human Monocyte Glucose Metabolism in Lymphoma. *J. Lab. clin. Med.*, **89**, 316.

NYDEGGER, N. E., ANNER, R., GEREBTZOFF, A., LAMBERT, P. H. & MICSCHER, P. A. (1973) Polymorphonuclear Leukocyte Stimulation by Immune Complexes. Assessment by Nitroblue Tetrazolium Reduction. *Eur. J. Immun.*, **3**, 465.

NYHOLM, R. E. & CURRIE, G. A. (1978) Monocytes and Macrophages in Malignant Melanoma. II. Lysis of Antibody-coated Erythrocytes as an assay of monocyte function. *Br. J. Cancer*, **37**, 339.

PARK, B. H., FIKRIG, S. M. & SMITHWICK, E. M. (1968) Infection and Nitroblue Tetrazolium Reduction by Neutrophils. A Diagnostic Aid. *Lancet*, ii, 532.

PICKERING, L. K., ANDERSON, D. C., SUNG CHOI & FEIGIN, R. D. (1975) Leukocyte Function in Children with Malignancies. *Cancer*, **35**, 1365.

RHODES, J. (1977) Altered Expression of Human Monocyte Fc Receptors in Malignant Disease. *Nature*, **265**, 253.

ROCKLIN, R. C., WINSTON, C. T. & DAVID, J. R. (1974) Activation of Sensitized Lymphocytes. *J. clin. Invest.*, **53**, 559.

ROSSEN, R. D., REISENBERG, M. D., HERSH, E. M. & GUTTERMAN, J. U. (1977) The Clq Binding Test for Soluble Immune Complexes: Clinical Correlations Obtained in Patients with Cancer. *J. natn. Cancer Inst.*, **58**, 205.

Segal, A. W. & Levi, A. J. (1973) The Mechanism of the Entry of Dye into Neutrophils in the Nitroblue Tetrazolium (NBT) Test. *Clin. Sci. mol. Med.*, **45**, 817.

Segal, A. W. & Levi, A. J. (1975) Factors Influencing the Entry of Dye into Neutrophil Leukocytes in the Nitroblue Tetrazolium Test. *Clin. Sci. mol. Med.*, **48**, 201.

Segal, A. W. & Peters, T. J. (1975) The Nylon Column Dye Test: a Possible Screening Test for Phagocytic Function. *Clin. Sci. mol. Med.*, **49**, 591.

Silverman, E. M. & Reed, R. E. (1973) The Nitroblue Tetrazolium Test in Lymphoma. *Am. J. clin. Path.*, **60**, 198.

Wenger, M. E. & Bole, C. G. (1973) Nitroblue Tetrazolium Reduction by Peripheral Leukocytes from Rheumatoid Arthritis and Systemic Lupus Erythematous Patients Measured by a Histochemical and Spectrophotometric Method. *J. Lab. clin. Med.*, **83**, 513.

Weston, W. L., Dustin, R. D. & Hecht, S. K. (1975) Quantitative Assay of Human Monocyte-Macrophage Function. *J. Immunol. Meth.*, **8**, 213.

Yam, L. T., Li, C. Y. & Crosby, W. H. (1971) Cytochemical Identification of Monocytes and Granulocytes. *Am. J. clin. Path.*, **55**, 283.